# Intestinal *Candida albicans* Promotes Hepatocarcinogenesis by Up-Regulating NLRP6

**DOI:** 10.3389/fmicb.2022.812771

**Published:** 2022-03-08

**Authors:** Zherui Liu, Yinyin Li, Chen Li, Guanglin Lei, Lin Zhou, Xiangling Chen, Xiaodong Jia, Yinying Lu

**Affiliations:** ^1^Peking University 302 Clinical Medical School, Beijing, China; ^2^Senior Department of Hepatology, Fifth Medical Center of Chinese PLA General Hospital, Beijing, China; ^3^Senior Department of Oncology, Fifth Medical Center of Chinese PLA General Hospital, Beijing, China

**Keywords:** *Candida albicans*, ITS, hepatocellular carcinoma, metabolome, NLRP6

## Abstract

Hepatocellular carcinoma (HCC), a primary liver cancer, is closely associated with the gut microbiota. However, the role of gut fungi in the development of HCC remains unclear. The aim of this study was to explore the influence of intestinal *Candida albicans* on HCC. Here, We found that patients with HCC showed significantly decreased diversity of the gut mycobiome and increased abundance of *C. albicans*, compared to the patients with liver cirrhosis. The gavage of *C. albicans* in the WT models increased the tumor size and weight and influenced the plasma metabolome, which was indicated by alterations in 117 metabolites, such as L-carnitine and L-acetylcarnitine, and several KEGG enriched pathways, such as phenylalanine metabolism and citrate cycle. Moreover, the expression of nucleotide oligomerization domain-like receptor family pyrin domain containing 6 (NLRP6) in the intestinal tissues and primary intestinal epithelial cells of the WT mice interacted with *C. albicans* increased. Notably, the colonization of *C. albicans* had no effect on tumor growth in *Nlrp6*^–/–^ mice. In conclusion, the abnormal colonization of *C. albicans* reprogrammed HCC metabolism and contributed to the progression of HCC dependent on NLRP6, which provided new targets for the treatment of HCC.

## Introduction

Hepatocellular carcinoma (HCC) is the fourth leading cause of cancer mortality worldwide (Yu and Schwabe, 2017). Owing to the asymptomatic nature of the early stages and limited treatment options, the global burden of HCC is steadily increasing (Ferlay et al., 2015; Yang et al., 2019). The mechanism behind hepatocarcinogenesis still requires further investigation. The gut microbiota, also known as the microflora, is considered the most important microecosystem in the human body, consisting of bacteria, archaea, viruses, and fungi (Eckburg et al., 2005; Li et al., 2019). In addition to playing a role in host metabolism and immunity, gut microbiota also contributes to a variety of liver diseases (Wu L. et al., 2021). In general, studies on gut microbiota and HCC emphasize bacteria while neglecting fungi because of their decreased abundance (Huffnagle and Noverr, 2013). However, accumulating evidence has shown that gut fungi have a significant influence on host health. Intestinal fungi affect not only the conditions of the gut but also the functions of other extraintestinal organs, including the liver (Wu X. et al., 2021). Several liver diseases such as alcoholic liver disease, non-alcoholic fatty liver disease, and cirrhosis have been closely associated with dysbiosis of the gut fungi (Kim et al., 2017; Yang et al., 2017). However, the specific role of gut fungi in HCC remains poorly characterized.

The ascomycete yeast *Candida albicans*, an opportunistic fungal pathogen, is the most prominent fungus inhabiting the human gut (Gow and Yadav, 2017). Since *C. albicans* resides together with hundreds of other microbial taxa, it is acknowledged that the gut microbiome influences *C. albicans* proliferation. However, *C. albicans* may switch to pathogenic mode when the host is immunosuppressed or has microbial dysbiosis (Perlroth et al., 2007; Fan et al., 2015). In general, *C. albicans* infections usually occur in the gastrointestinal tract, and many studies have revealed that *C. albicans* can enter the bloodstream by translocating through the intestinal barrier (Allert et al., 2018; Zhai et al., 2020) and has been closely related to the progression of several serious systemic diseases, including cancer (Ramirez-Garcia et al., 2016). It has been reported that systemic infection with *C. albicans* could promote the progression of breast cancer by dysregulating the cytokine network and regulatory T cells (Ahmadi et al., 2019). *C. albicans* has also been demonstrated to be involved in the promotion of gastric cancer (Zhong et al., 2021). Moreover, *C. albicans* was demonstrated to be associated with alcoholic hepatitis by the inflammatory IL-1β and its peptide toxin candidalysin (Yang et al., 2017; Chu et al., 2020). Although the role of *C. albicans* has been demonstrated in several liver diseases, the relationship between *C. albicans* and HCC remains elusive.

The nucleotide oligomerization domain-like receptor family pyrin domain containing 6 (NLRP6) plays a role in recognizing microbe-associated molecular patterns in the body and protects the host against pathogenic bacteria and viruses (Zheng et al., 2021). NLRP6, mainly expressed in the intestine, affects intestinal microbiota composition, which is community dependent and manifests when exposed to a sufficiently diverse microbiota configuration (Elinav et al., 2011; Levy et al., 2015). Studies performed in animals have demonstrated that NLRP6 acts as a tumor suppressor gene in several types of cancer such as colorectal (Hu et al., 2013), lung (Gao et al., 2019), and gastric cancer (Wang et al., 2020). It is unknown whether there is an association between NLRP6, HCC, and gut fungi. To investigate this association, we characterized the gut fungi of patients with HCC by internal transcribed spacer (ITS) sequencing, and two syngeneic HCC models of wild-type (Farshidfar et al., 2017) and Nlrp6^–/–^ mice with abnormal colonization of *C. albicans* were constructed in this study.

## Materials and Methods

### Participant Information and Stool Sample Collection

Seventeen patients with HCC (HCC group) and 11 patients with LC (LC group) were identified by pathological diagnosis for the first time. Those who received treatment with antibiotics or probiotics one-two months before sample collection were excluded. A stool sample was collected from each subject at the time of recruitment and stored at –80°C. The study was approved by the Ethics Committee of the Fifth Medical Center of Chinese PLA General Hospital. All participants provided written informed consent.

### Internal Transcribed Spacer Amplification and Bioinformatic Analysis

DNA extraction was performed based on the instructions of PowerSoil DNA Isolation Kit (#12888-100, MoBio, CA, United States). Isolated DNA was dissolved in Tris-EDTA buffer and stored at –80°C before use. The ITS1F/ITS1R primer pair (ITS1F: 5′-CTTGGTCATTTAGAGGAAGTAA-3′; ITS1R: 5′-GCTGCGTTCTTCATCGATGC-3′) were used to amplify the ITS region (Zhang et al., 2020). The amplification products were purified and quantified prior to library pooling. An Illumina MiSeq platform (Promegene Co. Ltd., Shenzhen, China) was used to sequence the libraries. Paired-end sequencing data from Promegene company were clean amplicons without barcodes and primers. The paired-end reads were merged and duplicated using VSEARCH (Version 2.14.1). UNOISE3 was used to denoise the dereplicated amplicon sequence variants. The feature table was produced using VSEARCH, and fungal sequences were identified using UNITE database. The α-diversity and β-diversity were created using USEARCH (Version 10.0.240) and R software (Version 3.6.1). Linear discriminant analysis effect size (LEfSe) was constructed using the ImageGP, with LDA score > 2.0 as an inclusion. STAMP software (Version 2.1.3) was used to compare the abundance at the species level.

### Strain Culture

*Candida albicans* SC5314 standard strain was purchased from Biofeng Co., Ltd. (Shanghai, China) and cultured on yeast extract-peptone-dextrose (YPD; TaKaRa, Beijing, China) agar plates for 48 h at 30°C. On the day before administration to mice, *C. albicans* was cultured overnight in sterilized YPD medium at 30°C (200 rpm). Cultures were centrifuged at 800 × *g* for 5 min. The supernatant was removed and washed twice with sterile PBS, adjusting *C. albicans* solution to a final concentration of 4 × 10^8^ colony-forming units (CFUs)/mL.

### Cell Culture

Hepatocellular carcinoma cell line Hepa1-6 was obtained from the Fifth Medical Center of Chinese PLA General Hospital. The cells were cultured in Dulbecco’s modified Eagle medium (Thermo, Waltham, MA, United States) with 10% fetal bovine serum (Gibco, Grand Island, NY, United States) and 1% penicillin-streptomycin in a humidified atmosphere of 5% CO_2_ at 37°C.

### Animal Experiments

Male C57BL/6 mice (4 weeks old) were purchased from SPF Biotechnology (Beijing, China), housing under the special pathogen free (SPF) animal lab at the Fifth Medical Center of Chinese PLA General Hospital at a temperature of 20–26°C, 50% relative humidity, and a 12/12 h light-dark cycle. Food and water were provided *ad libitum*. After one week of acclimatization, mice were switched to antibiotic cocktail (ABX; containing 1 mg/mL neomycin, 1 mg/mL bacitracin, and 1 mg/mL streptomycin) until the end of the experiment to maintain the gut in a favorable state for *C. albicans* colonization (Jiang et al., 2017). Solutions and bottles were changed every 2–3 days. After one week of ABX pretreatment, the mice were randomized to *C. albicans* and control groups. The *C. albicans* group was administered *C. albicans* by oral gavage at a dose of 2 × 10^8^ CFUs in sterile PBS (0.5 mL), and the control group was administered sterile PBS (0.5 mL) as a control. The gavage was conducted every other day for 3 weeks. Efficient colonization was checked by culture of feces on a chromogenic Candida agar plate (HuanKai Microbiotal, Guangzhou, China) one week post oral gavage. After *C. albicans* colonization, 5 × 10^6^ Hepa1-6 cells were inoculated subcutaneously on the left flank of the mice, and the mice were euthanized 2 weeks later. Tumors were isolated and weighed, the tumor volume was calculated as (tumor length × tumor wide^2^) × 0.52 (Erkes et al., 2020). Blood plasma samples and intestine tissues from WT mice were collected and immediately stored at –80°C before metabolomic and RNA expression analysis. Moreover, age-and sex-matched *Nlrp6*^–/–^ mice (a generous gift from Grace Y. Chen; Comprehensive Cancer Center, Ann Arbor, MI, United States; Chen G. Y. et al., 2011) were used, and the experiment was conducted using the same experimental protocol. All animal studies were approved by the Animal Welfare and Ethics Committee of the Fifth Medical Center of the PLA General Hospital.

### Collection and Preparation of Blood Plasma

At the end of the experiment, blood samples were collected into sterile frozen 1.5 mL eppendorf tubes, with EDTA as anticoagulant. After centrifugation at 3,000 rpm for 15 min at 4°C, supernatants were collected to obtain plasma. Fifty milligrams of each blood plasma sample was weighed into a 1.5 mL eppendorf tube and 1000 μL of extract solvent (acetonitrile-methanol-water, 2:2:1, containing 20 μL internal standard; CNW Technologies) was added to all samples. After 30 s vortexing, the mixture was homogenized three times at 45 Hz for 4 min, and sonicated for 5 min under 45 Hz frequency in an ice-water bath. Homogeneity was then incubated at –20°C for 1 h, followed by centrifugation at 12,000 rpm for 15 min at 4°C. The obtained supernatants were transferred to LC-MS vials and stored at –80°C until analysis.

### Liquid Chromatographytandem Mass Spectrometry Analysis

The Liquid Chromatographytandem Mass Spectrometry (LC-MS/MS) analysis was performed using an Agilent 1290 UHPLC system combined with a Q Exactive Orbitrap mass spectrometer (Thermo Fisher Scientific, United States). The MS system was set in positive ion mode. The spray voltage and capillary temperatures were 3.8 kV and 320°C. The mass scanning range was set at 70–1000 m/z at a speed of 7 Hz. Full MS resolution, MS/MS resolution, sheath gas and Aux gas flow rate were set at 70,000, 17,500, 45 Arb, and 15 Arb, respectively. Collision energy in the NCE model was set to 20/40/60 eV. Mobile phase A was 0.1% (v/v) formic acid, and mobile phase B was acetonitrile. The gradient program was as follows: 0 - 1 min, 1% B; 8 min, 99% B; 10 min, 99% B; 10 min, 1% B; 12 min, 1% B. The flow rate was set to 0.5 mL/min, and the sample injection volume was 2 μL. Xcalibur software (Version 4.0.27, Thermo) was used to obtain MS data and identify as many metabolites as possible. The MS raw data were transformed to mzML format, and processed by R package XCMS (version 3.2). The data were filtered using the XCMS program. Then, each metabolite was normalized with the internal standard, missing values were imputed by semi-minimum values for a feature. Principal component analysis (PCA) and orthogonal partial least squares-discriminant analysis (OPLS-DA) were performed by R packages after data preprocessing and annotation procedures. The differential metabolites were screened by using variable importance in projection (VIP) score of the OPLS model combined *t*-test. The threshold for screening was VIP ≥ 1 and *p* < 0.05. Finally, enrichment analysis of the significant signal transduction pathways based on the differential metabolites was carried out using KEGG.

### Isolation and Infection of Primary Intestinal Epithelial Cells

A modified method previously described was applied for isolation of IECs (Ren et al., 2017). Six WT mice purchased from SPF Biotechnology (Beijing, China) were euthanized. The intestines were isolated and the ileum was removed and flushed with ice-cold PBS. The ileum was cut longitudinally and rinsed in ice-cold PBS. The entire ileum was then cut into 1 mm^3^ fragments, total fragments were transferred to centrifuge tubes and washed three times in PBS at 50 × *g* for 3 min, followed by incubation in washing medium containing collagenase type I and hyaluronidase for 25 min at 37°C. The samples were allowed to stand for 1 min at 25°C. The supernatant was removed following centrifugation at 100 × *g* for 5 min. After suspension in complete IEC medium, primary IECs were harvested by centrifugation three times at 100 × *g* for 5 min. Cells were suspended in complete IEC medium and seeded in polylysine-coated culture dishes (Procell, Wuhan, China) in 5% CO_2_ at 37°C. After 90 min, non-adherent cells were transferred into new rat tail tendon collagen-coated 24-well plates (Solarbio, Beijing, China) to a density of 1 × 10^6^ IECs/well. The IECs were infected with *C. albicans* or complete medium at a multiplicity of infection of 1 for 6 h. The culture supernatant was removed and the plates were rinsed three times in PBS. The IECs were collected by centrifugation at 250 × *g* for 10 min and stored at –80°C for further analysis.

### Gene Expression Analysis

Total RNA from intestinal tissues and IECs were separately isolated based on the manufacturer’s instruction of RNA isolation kit (R1200, Solarbio, Beijing, China). cDNA was synthesized using a cDNA synthesis Mix Kit (KR118, TIANGEN, Beijing, China). Quantitative real-time polymerase chain reaction was performed using SYBR Green qPCR PreMix (FP207, TIANGEN, Beijing, China). *Nlrp6* gene expression was normalized to that of β-actin. The ΔΔCT method was used for *Nlrp6* gene expression analysis. The primer sequences used were: forward primer *Nlrp6* 5′-CTGGCGTCATTGTGGAACCTCT-3′ and the reverse primer *Nlrp6* 5′-TCTCACTCAGCTCCACAGAGGT-3′; forward primer β-actin 5′-CATTGCTGACAGGATGCAGAAGG-3′ and the reverse primer β-actin 5′-TGCTGGAAGGTGGACAGT GAGG-3′.

### Statistical Analysis

Statistical analysis was performed using SPSS 22.0 software and GraphPad Prism (GraphPad Software, San Diego, CA, United States) and R 3.6.2. In all statistical analyses, *p* values < 0.05 were considered significant.

## Results

### The Diversity of Gut Fungi in Patients With Hepatocellular Carcinoma Are Altered

**FIGURE 1:**
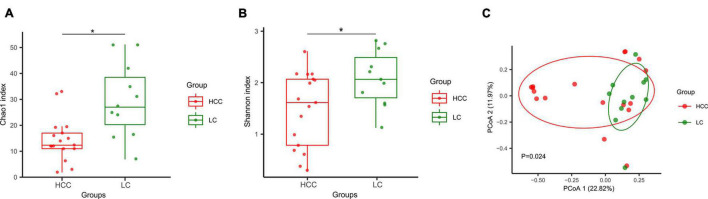
Composition and diversity of gut fungi between patients with hepatocellular carcinoma (HCC group *n* = 17) and liver cirrhosis (LC group *n* = 11). **(A,B)** α-diversity was measured by Chao1 and Shannon indexes. Boxplots display the median with interquartile range, *p < 0.05. Groups were compared using an unpaired *t* test. **(C)** β-diversity was measured by Bray–Curtis distance. Each dot represents one sample. *P* = 0.024, permutational multivariate analysis of variance by Adonis. *p* < 0.05 considered significant.

**FIGURE 2:**
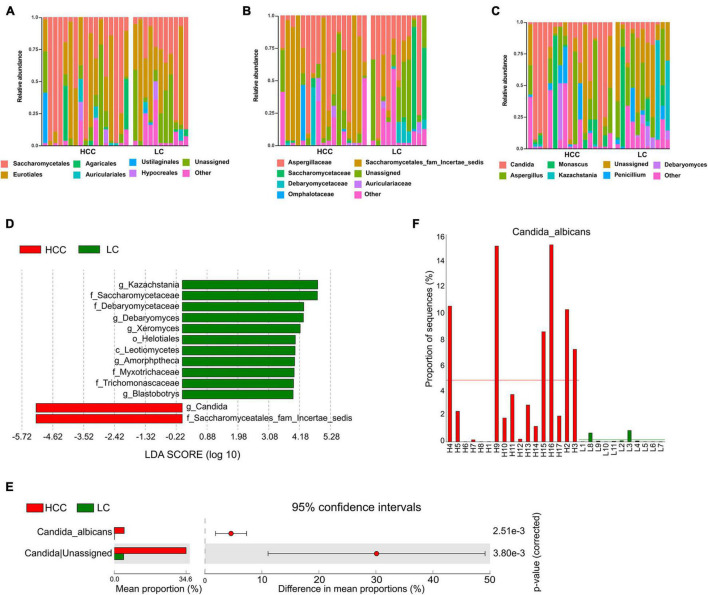
Differentially abundant taxa in patients with hepatocellular carcinoma (HCC) compared to patients with liver cirrhosis (LC). Relative abundance plots of fungal taxa at **(A)** order, **(B)** family, and **(C)** genus level. **(D)** The histogram represents linear discriminant analysis (LDA) scores of bacteria with significant differential abundance (LDA > 2) between the compared groups, as represented by different colors. The taxa (LDA > 2) are shown. **(E)** Differentially abundant fungal species between HCC and LC groups. Groups were compared using Welch’s *t* test. **(F)** Abundance of *C. albicans* in each individual. Taxa differences are shown with *p* < 0.05.

Stool samples from 11 patients with liver cirrhosis (LC; LC group) and 17 patients with HCC (HCC group) were collected for ITS sequencing. The detailed clinical characteristics of all patients are displayed in Supplementary Table 1. We then compared the fungal diversity between the two groups. The α-diversity based on Chao1 and Shannon indices showed that the HCC group had lower biodiversity than the LC group (Figures 1A,B). To display the gut mycobiome space between the two groups, β-diversity was calculated according to the Bray–Curtis distance. Principal coordinate analysis results showed that the HCC and LC groups aggregated separately, suggesting that the gut fungal community had a different distribution in the HCC and LC groups (Figure 1C). Moreover, the fungal profiling was performed for each subject to identify the main taxon at the order, family and genus level (Figures 2A–C). These results reveal that the composition of gut fungi in patients with HCC is significantly altered compared to that in patients with LC.

### *Candida albicans* Significantly Increases in the Gut of Patients With Hepatocellular Carcinoma

Linear discriminant analysis effect size analysis was applied to identify the major differential fungi between LC and HCC. The results showed that at the family level, patients with LC showed a higher abundance of *Myxotrichaceae*, *Debaryomycetaceae*, *Trichomonascaceae*, and *Saccharomycetaceae*. However, the family *Saccharomycetales fam Incertae sedis* was more abundant in patients with HCC than in the LC group. At the class level, *Leotiomycetes* were more abundant in the LC group. At the genus level, *Kazachstania*, *Debaryomyces*, *Xeromyces*, *Amorphotheca*, and *Blastobotrys* were more enriched in the LC group. The genus *Candida* was significantly overrepresented in HCC patients (Figure 2D). We also evaluated fungal alterations at the species level using STAMP. According to the results of Welch’s *t*-test, *C. albicans* (*p* < 0.001) was significantly increased in the HCC group (Figure 2E). The abundance of *C. albicans* was further evaluated for each participant in the two groups. The results confirmed that *C. albicans* was more abundant in the HCC group than in the LC group (Figure 2F). These results demonstrated that *C. albicans* was significantly elevated in the HCC group.

### *Candida albicans* Promotes the Progression of Hepatocellular Carcinoma

**FIGURE 3:**
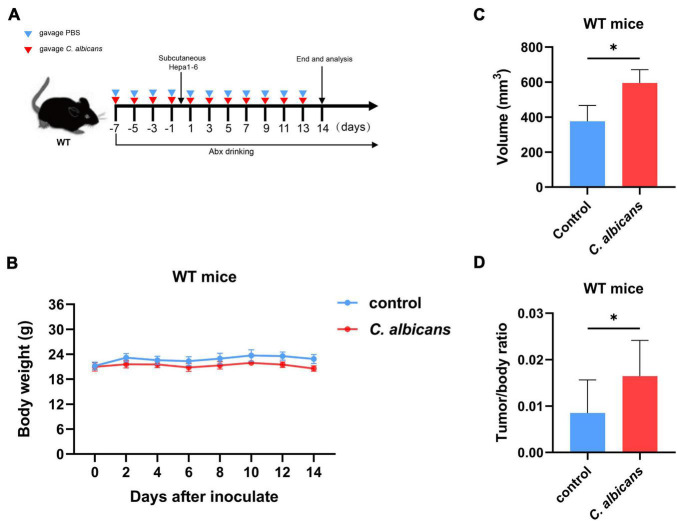
Abnormal colonization of *Candida albicans* in the gut promotes hepatocellular carcinoma development. **(A)** Schematic diagram of the oral gavage intervention protocol utilizing phosphate-buffered saline (PBS; Control group *n* = 7) or *C. albicans* (*C. albicans* group *n* = 7) to C57BL/6 mice. **(B)** Growth of weights in C57BL/6 mice with gavage of PBS or *C. albicans*. Body weights were measured at the indicated time points. **(C,D)** Tumor volumes and tumor weight/body weight at the experimental endpoints. Groups were compared with student’s *t*-test. Values are mean ± SD, *p < 0.05.

To determine the association between intestinal *C. albicans* and HCC, we used C57BL/6 mice with oral gavage of *C. albicans* [oral gavage of sterile phosphate-buffered saline (PBS) as control] from one week prior to Hepa1-6 inoculation (Figure 3A). The results of gut colonization by *C. albicans* are shown in Supplementary Figure 1. During the 14 days of body weight monitoring, we found that the body weights of the two groups were not significantly different (Figure 3B). However, the tumor volume and tumor weight/body weight were significantly increased in the *C. albicans* group at the end of the experiment (Figures 3C,D). These results reveal that abnormal colonization by *C. albicans* contributes to the growth of liver tumors.

### Intestinal Colonization of *Candida albicans* Reprograms the Metabolome of Blood Plasma

**FIGURE 4:**
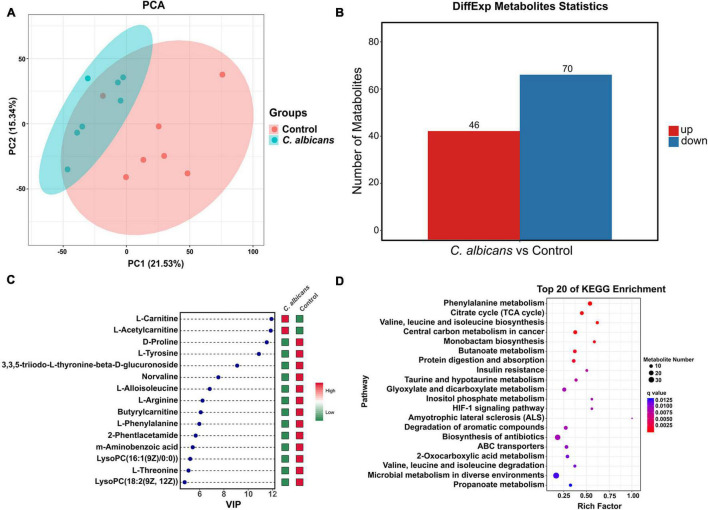
Intestinal colonization of *Candida albicans* reprograms the metabolome of blood plasma. **(A)** PCA score plots based on the LC-MS/MS data of all samples from two groups (Control group *n* = 7; *C. albicans* group *n* = 7). Shaded areas are the 95% confidence regions of each group. PC1 and PC2 represent the first two main components, and they reflect the contribution to the sample difference, expressed as a percentage. **(B)** Statistical chart of different metabolites between two groups. All of the shown metabolites are statistically different (The threshold of VIP was set to 1 and *p* < 0.05 as determined by univariate *t* test). **(C)** Variable Importance in Projection (VIP) score plot of the top 15 metabolites that differed in Control group vs. *C. albicans* group. **(D)** Top 20 of KEGG enrichment bubble chart. The bubble size indicates the quantity; the redder the color, the smaller the *P*/*Q* value.

To investigate the association between the abnormal colonization of *C. albicans* and blood plasma metabolites in the host, we used non-targeted metabolomics to profile blood plasma from *C. albicans* and control groups in WT mice. We performed PCA analysis, an unsupervised multi-dimensional statistical analysis method, to analyze the blood plasma metabolic profiles in the two mice groups. The results showed that the samples of the two groups were aggregated separately (Figure 4A). The results indicated that the blood plasma metabolites of the *C. albicans* group showed pronounced metabolic alterations that were different from those of the control group to some extent. To screen for significant differences in metabolites between the two groups, we co-analyzed the VIP value (≥1) from OPLS-DA analysis and the *p* value (<0.05) from the *t*-test. According to the results, 46 upregulated metabolites and 70 downregulated metabolites that differed in abundance were identified in the *C. albicans* group compared to the control group (Figure 4B). The metabolites were sorted by VIP values to screen for the most important compositions. As shown in Figure 4C, L-carnitine and L-acetylcarnitine had higher VIP values than the other metabolites, and increased in the *C. albicans* group, whereas the D-proline, L-tyrosine, L-arginine, etc. were decreased. We further analyzed the KEGG metabolic pathways associated with the abnormal colonization of *C. albicans* in HCC. These pathways are mainly related to phenylalanine metabolism, citrate cycle (TCA cycle), central carbon metabolism in cancer, arginine and proline metabolism, valine, leucine, and isoleucine biosynthesis and degradation, and the Hypoxia-inducible factor 1 signaling pathway (Figure 4D). In summary, our results revealed that the abnormal colonization of *C. albicans* changed plasma metabolism, involving metabolites and the corresponding signaling pathway.

### NLRP6 Plays an Essential Role in Promoting Hepatocellular Carcinoma During the Abnormal Colonization of *Candida albicans*

**FIGURE 5:**
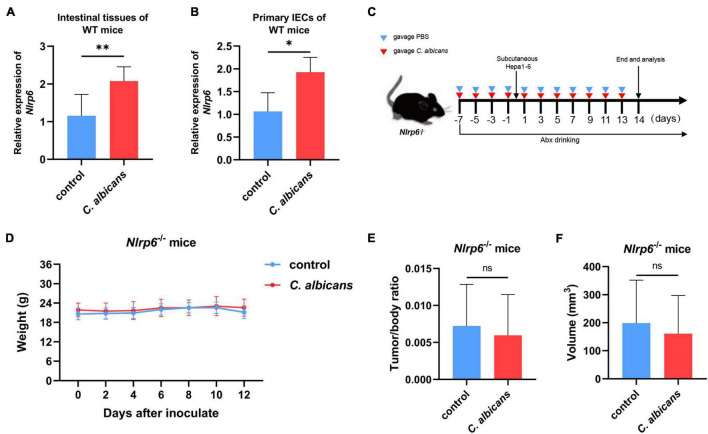
NLRP6 mediates the promotion of HCC by *Candida albicans*. **(A)** Nlrp6 expression levels in two groups of WT mice intestinal tissues (Control group *n* = 7, *C. albicans* group *n* = 7). **(B)** Nlrp6 expression levels in primary intestinal epithelial cells (IECs) from WT mice *in vitro* (Control group *n* = 3, *C. albicans* group *n* = 3). **(C)** Schematic diagram of the oral gavage intervention protocol utilizing phosphate-buffered saline (PBS; Nlrp6^–/–^ Control group *n* = 10) or *C. albicans* (Nlrp6^–/–^ *C. albicans* group *n* = 10) to Nlrp6^–/–^ mice. **(D)** Growth of weights in Nlrp6^–/–^ mice with PBS or *C. albicans*. Body weights were measured at the indicated time points. **(E,F)** Tumor volumes and tumor weight/body weight at the oral administration endpoints. Values are mean ± SD, *p < 0.05, ^**^p < 0.01 as determined by unpaired Student’s *t*-test.

NLRP6 has been reported to regulate host defense against microbes (Levy et al., 2015; Li and Zhu, 2020). Therefore, in order to investigate whether NLRP6 regulates host defense during the colonization of *C. albicans*, we detected the expression of *Nlrp6* in the intestines of WT mice with and without administration of *C. albicans*. The results of gut colonization by *C. albicans* were shown in Supplementary Figure 2. As shown in Figure 5A, the mRNA level of *Nlrp6* increased significantly in WT mice colonized by *C. albicans*. Then, the primary intestinal epithelial cells (IECs) isolated from WT mice were co-cultured with *C. albicans in vitro* to detect the expression of *Nlrp6*. As a result, *C. albicans* induced the expression of *Nlrp6* (Figure 5B). Moreover, *Nlrp6*^–/–^ mice were subjected to similar animal experiments as previously mentioned (Figure 5C) to explore the link between *Nlrp6* and colonization of *C. albicans* in HCC. There were no significant differences in body weight between the two *Nlrp6*^–/–^ groups during tumor-bearing (Figure 5D). At the end of the experiment, the tumor size and the ratio of tumor weight and body weight of the two *Nlrp6*^–/–^ mice were also not significantly different (Figures 5E,F). This is a surprising finding that *Nlrp6* is essential for promoting HCC caused by the abnormal colonization of *C. albicans*.

## Discussion

In this study, we report three major findings. Patients with HCC had decreased biodiversity and differential compositions of gut fungi compared to patients with LC. Administration of *C. albicans* promoted tumor growth in the WT model but not in the *Nlrp6*^–/–^ model. In addition, *C. albicans* reprogrammed the plasma metabolome of the WT model.

It is well known that commensal fungi are potentially involved in enteric disease such as colitis, Crohn’s disease, and inflammatory bowel disease (IBD) (Sokol et al., 2017; Imai et al., 2019), and extraintestinal diseases including liver diseases. Chen Y. et al. (2011) have found that the alteration of gut fungi is associated with the progression and severity of the disease in patients with hepatitis B virus. Additionally, exposure to fungal products such as curdlan, paramylon, and zymosan in hepatic macrophages can promote the progression of alcoholic liver disease (Yang et al., 2017). However, the mechanistic role of gut fungi in HCC has not been fully explored. To the best of our knowledge, this is the first study to explore the relationship between gut fungi and HCC. In this study, we analyzed the ITS sequences of the stools of patients with HCC and patients with LC to explore the composition and ecological alterations of fungi associated with HCC. According to our results, patients with HCC showed lower α- and β-diversity than patients with LC. Evidence has shown that IBD patients have lower α-diversity compared with healthy individuals (Sokol et al., 2017). Furthermore, a lower diversity of gut fungi has also been observed in patients with alcoholic liver disease (Lang et al., 2020). These characteristics are similar to the results of the present study. Inversely, the diversity of gut fungi was higher in the more severe type of patients with chronic HBV infection (Chen Y. et al., 2011). Thus, the relation of gut fungal diversity with liver diseases needs further clarification, although it has been reported that a decreased microbial diversity is often related to poor clinical outcomes (Malard et al., 2018).

*Candida albicans* is a normal commensal in the human body and causes no damage (Ramirez-Garcia et al., 2016). However, it can shift from commensal to pathogen when host defenses are weakened or individuals with inborn errors of immunity (Puel et al., 2010). Studies have shown that overgrowth of *C. albicans* on the mucosal epithelium is related to the production of carcinogens and the metabolism of pro-carcinogens (Ramirez-Garcia et al., 2016). It has been reported that *C. albicans* disorders are associated with several types of cancer, such as colorectal, oral, and pancreatic cancer (Kazmierczak-Siedlecka et al., 2020). In these situations, *C. albicans* induces hematogenous dissemination and spreads to extraintestinal organs, causing serious problems (Nobile and Johnson, 2015). In the liver, studies have revealed that *C. albicans* overgrowth promotes damage to hepatocytes and the development of ethanol-induced liver disease through increased IL-1β expression and secretion (Yang et al., 2017). Candidalysin, an exotoxin secreted by *C. albicans*, has also been shown to increase in patients with alcoholic hepatitis and to exacerbate ethanol-induced liver disease by CLEC7A signaling on bone marrow-derived cells in mice (Chu et al., 2020). However, the relationship between *C. albicans* and HCC has not yet been elucidated. Our study showed an increased abundance of *C. albicans* in HCC patients compared with LC patients for the first time, suggesting that *C. albicans* might play a potential role in the progression of HCC. To further confirm this result, we performed an HCC murine model of *C. albicans* colonization. Because the competing microbiota plays a significant role in the colonization of *C. albicans*, pretreatment with ABX is necessary to ensure successful colonization (Jiang et al., 2017). The tumor volume and tumor weight/body weight ratio were significantly increased in the *C. albicans* group of WT mice as compared to the control group, which confirms our speculation based on the data obtained from the patients with HCC in the clinic. However, it should be noted that, according to our results, an unassigned species of the genus *Candida* with significantly increased abundance in HCC group could not be further explored as it has not been identified. The database for the molecular identification of fungi and culturomic techniques requires further improvement.

Gut microbiota can affect host metabolism, including blood metabolites (Pedersen et al., 2016; Visconti et al., 2019). Therefore, in the present study, we performed LC-MS/MS analysis to further explore the influence of intestinal colonization by *C. albicans* on the plasma metabolome. The results of PCA analysis indicated a separation of plasma metabolic conditions between control and *C. albicans* groups in WT mice, suggesting that *C. albicans* colonization does change the metabolome in WT mice to some extent. Our results further revealed that 46 metabolites were significantly upregulated and 70 metabolites were significantly downregulated in the *C. albicans* group compared with the control group. Moreover, we identified specific metabolites involved in the mentioned phenotype. According to the results of the VIP score, we found that L-carnitine and L-acetylcarnitine were significantly higher in the *C. albicans* group than in the control group. A limitation of our study is that we did not confirm the levels of these two metabolites in the blood plasma of clinical HCC patients. Moreover, several studies have reported that the concentrations of L-carnitine and L-acetylcarnitine differentiate patients with HCC from those with liver diseases or health controls (Zhou et al., 2012; Fujiwara et al., 2018). However, Chen et al. found significantly increased levels of serum long-chain acylcarnitines and decreased levels of medium-chain acylcarnitines in patients with HCC compared with health control (Chen et al., 2013). It is known that long-chain acylcarnitines play an important role in the carnitine shuttle, which can transport long-chain fatty acids into the mitochondria for oxidation and further energy supply (McCann et al., 2021). Therefore, it may explain that an increased requirement of energy consumption in HCC patients results in long-chain acylcarnitines accumulation and activation of carnitine shuttle system for oxidation of long-chain fatty acids to supply more usable energy. Although the relationship among L-carnitine, L-acetylcarnitine, and HCC needs further study, our results showed an altered carnitine metabolism during abnormal colonization of *C. albicans* in mice. Studies have shown that carnitine is mediated by carnitine palmitoyltransferase through the cell membrane and mitochondrial membrane layer by layer, and its products finally enter the TCA cycle (McCann et al., 2021). The high concentration of blood plasma L-carnitine causes a more activated TCA cycle, which could act as a fuel for tumor proliferation (Vander Heiden and DeBerardinis, 2017). Our results also revealed alterations in the TCA cycle during abnormal colonization by *C. albicans*, which is in accordance with the findings of previous studies. Although there exists “Warburg effect” in cancer cells, it was confirmed that mitochondrial metabolism also plays an important role in cancer cell growth (DeBerardinis and Chandel, 2020). However, it has been also reported that the carnitine might exert preventive effects on HCC development. Ishikawa et al. (2014) has found that administration of L-carnitine in mice can prevent the progression of non-alcoholic steatohepatitis and further inhibit liver carcinogenesis by suppressing oxidative stress and inflammation in the liver. Therefore, relevant relationships and potential signal pathways between L-carnitine and the progression of HCC should be further studied. Moreover, results based on the VIP score also showed that the levels of D-proline and L-tyrosine was significantly decreased in *C. albicans* group compared with control group. Rocha et al. reported that patients with lung cancer has decreased level of tyrosine in plasma compared with health subjects by using NMR-Based metabonomics (Rocha et al., 2011). Norton et al. (1985) found a decreased level of plasma tyrosine in patients with esophageal cancer and proline in patients with lymphoma, esophageal cancer, osteosarcoma and soft-tissue sarcoma compared with health control. What we found is in line with those previous studies. However, most current studies on proline and tyrosine are focused on the screening and diagnosis of cancer. The role of proline and tyrosine in HCC progression has not been fully explained, remains further explored. Furthermore, it has been demonstrated that the phenylalanine metabolism pathway is altered in several types of cancers, such as gastric cancer and prostate cancer (Lario et al., 2017; Zhao et al., 2017). According to our results, an obvious change in phenylalanine metabolism was also observed, which is similar to the results of previous studies. Meanwhile, we noticed that some of the metabolites and pathways were also altered in our HCC models. In summary, our results provide evidence that abnormal colonization by *C. albicans* acts as an important effector of HCC metabolism and establishes a new correlation and potential mechanism between *C. albicans* and carnitine in progression of HCC. Previous studies have verified that host-microbe interactions contribute to various diseases, including HCC, through their impact on metabolism (Jee et al., 2018; Kurilshikov et al., 2019; Zhao et al., 2021). Reprogrammed metabolism is also a hallmark of cancer. The altered metabolome in this study provided new insight for understanding HCC, and the impact of the specific metabolites on the progression of HCC will be our next research work.

As a pattern recognition receptor, NLRP6 has been reported to play a role in protecting the host against pathogenic bacteria and viruses. Previous studies revealed that NLRP3, another subset of the NLR family, plays an important role in the recognition of *C. albicans* and further impacts on body health and diseases (Joly and Sutterwala, 2010). However, the relationship between NLRP6 and *C. albicans* remains unclear. Moreover, the link between intestinal dysbiosis and HCC has been well established; thus, it is also important to investigate the underlying mechanism of *C. albicans* colonization in HCC. As *NLRP6* is highly expressed in the intestine and is involved in maintaining intestinal homeostasis (Xue et al., 2019), we explored *Nlrp6* expression levels in intestinal tissues obtained from the WT HCC murine model in our animal experiments. We observed that the expression levels of *Nlrp6* were significantly higher in the *C. albicans* group than in the control group. Considering that the host response to pathogens is mainly induced by IECs at the gut-microbial interface, we further investigated the effect of *C. albicans* on primary IECs using an *in vitro C. albicans* infection model. It is interesting that the expression of *Nlrp6* was also increased in *C. albicans* compared with that in the control group, indicating that intestinal NLRP6 plays a role in the response to *C. albicans*. Although previous studies have reported that *C. albicans* can inhibit *NLRP3* and *NLRP6* expression in Caco-2 cells (Mao et al., 2020), it is important to note that since the Caco-2 cell line is derived from a human colon adenocarcinoma cell line, it could not fully reflect the real response to abnormal colonization by *C. albicans*. In contrast, primary IECs maintain many important signatures and functions of cells in the body. To further confirm our findings, we performed animal experiments using the same *C. albicans* intervention in *Nlrp6*^–/–^ mice. Interestingly, after *C. albicans* colonization, the tumor volume between the control and *C. albicans* groups in *Nlrp6*^–/–^ mice showed no significant difference. However, there is also a disadvantage in our study. The intestine-specific conditional *Nlrp6* knockout mice are better than the whole-body knockout of *Nlrp6* in the experiments. Therefore, the data from intestine-specific conditional *Nlrp6* knockout mice might be more persuasive. Moreover, it is also important to note that in addition to the initial stage of abnormal colonization of *C. albicans* in the IECs, hepatic endothelial cells also play a paramount role in response to *C. albicans*. It has been shown that *C. albicans* can stimulate synthesis of IL-18, TNF-α, IL-1α, and IL-1β in endothelial cells (Orozco et al., 2000), which could generate a hepatic pro-inflammatory microenvironment and induce the expression of E-selectin and vascular cell adhesion molecule-1, lead to cancer growth, adhesion and metastasis in the liver, such as hepatic melanoma (Vidal-Vanaclocha et al., 2000; Rodriguez-Cuesta et al., 2010; Ramirez-Garcia et al., 2013). Moreover, as the NLRP6 is an intracellular PRR, it is usually considered as a downstream of recognizing pathogen, that is, a mediator activated and stimulated the inflammation and host defense (Medzhitov, 2007; Anand et al., 2012). In actually, it has been reported that during the infection with *Candida*, the PRRs on the surface of phagocytes can recognize the fractions of *Candida* such as mannans, β-glucans, and chitin (Gazi and Martinez-Pomares, 2009; Netea and Marodi, 2010), and exert antifungal immunity through the Toll-like receptors (TLRs) including TLR2, TLR4, and C-type lectin receptors (CLRs) such as mannose receptor, dectin-1, dectin-2, and DC-specific intracellular adhesion molecule-grabbing non-integrin (Marodi et al., 1991; Tada et al., 2002; Brown, 2006). Therefore, another disadvantage of our study is that we did not explore the cell surface receptors which recognizes *C. albicans* and further activates the NLRP6. However, it is also exciting that our study establishes a link between *C. albicans* and *Nlrp6* in the progression of HCC for the first time, revealing that the detrimental effect of *C. albicans* on HCC may be achieved through the mediation of NLRP6.

In conclusion, we characterized the gut mycobiome of HCC patients and demonstrated that the abnormal colonization of *C. albicans* in the gut changed HCC metabolism and contributed to the progression of HCC depending on NLRP6, providing new targets for the treatment of HCC.

## Data Availability Statement

The datasets presented in this study can be found in online repositories. The raw sequence data reported in this paper have been deposited in the Genome Sequence Archive in National Genomics Data Center, China National Center for Bioinformation/Beijing Institute of Genomics, Chinese Academy of Sciences (GSA: CRA004907) that are publicly accessible at https://ngdc.cncb.ac.cn/gsa.

## Ethics Statement

The studies involving human participants were reviewed and approved by the Ethics Committee of the Fifth Medical Center of Chinese PLA General Hospital. The patients/participants provided their written informed consent to participate in this study. The animal study was reviewed and approved by the Animal Welfare and Ethics Committee of the Fifth Medical Center of the PLA General Hospital.

## Author Contributions

ZL conducted the experiments, executed most of the data processing and analysis, and wrote the manuscript. YLi, CL, GL, LZ, and XC conducted the experiments and analyzed the data. XJ and YLu participated in the designing of the experiments and data analysis and guided and supervised the work. All authors read and approved the submitted version.

## Conflict of Interest

The authors declare that the research was conducted in the absence of any commercial or financial relationships that could be construed as a potential conflict of interest.

## Publisher’s Note

All claims expressed in this article are solely those of the authors and do not necessarily represent those of their affiliated organizations, or those of the publisher, the editors and the reviewers. Any product that may be evaluated in this article, or claim that may be made by its manufacturer, is not guaranteed or endorsed by the publisher.
