# Supplementary material for: Intestinal Candida albicans Promotes Hepatocarcinogenesis by Up-Regulating NLRP6
Source: Front Microbiol. 2022 Mar 8;13:812771. doi: 10.3389/fmicb.2022.812771 (PMC8964356; doi:10.3389/fmicb.2022.812771)
Supplement: Supplementary file 1 [file Data_Sheet_1.docx]

**Supplementary Figures**

**
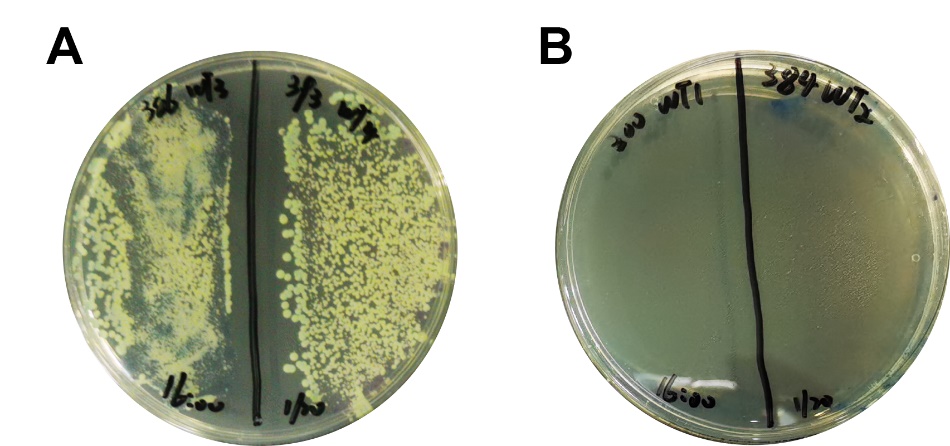
**

**Supplementary Figure 1.** The appearance of *Candida albicans* colonies in wild-type mice on Chromogenic Candida Agar. (A) Appearance in *C. albicans* group. (B) Appearance in control group. Greenish-blue-green colonies indicate colonization of *C. albicans*.


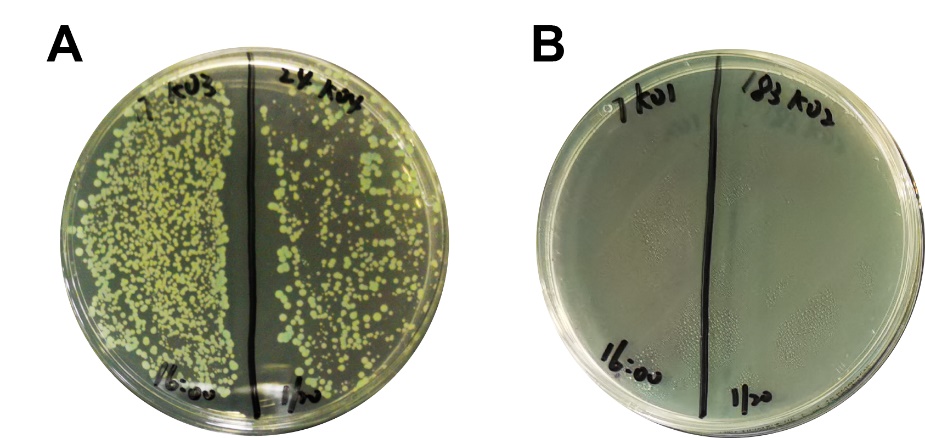


**Supplementary Figure 2.** The appearance of *Candida albicans* colonies in NLRP6^-/-^ mice on Chromogenic Candida Agar. (A) Appearance in *C. albicans* group. (B) Appearance in control group. Greenish-blue-green colonies indicate colonization of *C. albicans*.

**Supplementary Table 1.** Basic characteristics of the subjects in each HCC (hepatocellular carcinoma) and LC (liver cirrhosis) group.

| **Variable** | **LC (n=11)** | **HCC (n=17)** |
| --- | --- | --- |
| Sex (male/female) | 9/2 | 15/2 |
| Age (y) | 50.73 ± 6.08 | 55.82 ± 8.81 |
| AFP (ng/mL) |  |  |
| ≤20 | 11 (100%) | 8 (47.06%) |
| >20 | 0 (0%) | 9 (52.94%) |
| Tumor size (cm) |  |  |
| ≤2 | - | 2 (13.33%) |
| >2 | - | 15 (86.67%) |
| AST (U/L) | 26.09 ± 12.26 | 43.85 ± 19.78 |
| ALT (U/L) | 20.18 ± 10.97 | 33.12 ± 17.10 |
| ALP (U/L) | 93.82 ± 47.92 | 132.47 ± 54.19 |
| GGT (U/L) | 31.36 ± 19.72 | 162.65 ± 137.63 |
| TBIL (mg/dL) | 16.23 ± 10.68 | 17.39 ± 11.11 |
| DBIL (mg/dL) | 6.78 ± 4.73 | 8.95 ± 7.64 |
| Total protein (g/L) | 64.09 ± 4.74 | 64.88 ± 6.40 |
| Albumin (g/L) | 37.00 ± 5.16 | 35.53 ± 4.00 |
| Globulin (g/L) | 27.00 ± 3.22 | 28.46 ± 5.40 |

AFP, alpha-fetoprotein; AST, aspartate transaminase; ALT, alanine transaminase; ALP, alkaline phosphatase; GGT, gamma-glutamyl transpeptidase; TBIL, total bilirubin; DBIL, direct bilirubin.
